# Vulvar ulcers secondary to aortic angiosarcoma in a patient with misdiagnosed cancer of unknown primary origin^⋆^^☆^

**DOI:** 10.1016/j.abd.2024.07.008

**Published:** 2025-01-13

**Authors:** Denis Miyashiro, Regina Barros Domingues, Malu Viter da Rosa Barbosa, Claudia Giuli Santi, Marcello Menta Simonsen Nico, José Antonio Sanches

**Affiliations:** a Department of Dermatology, Faculdade de Medicina da Universidade de São Paulo, São Paulo, SP, Brazil; b Department of Pathology, Faculdade de Medicina da Universidade de São Paulo, São Paulo, SP, Brazil; c Instituto do Câncer do Estado de São Paulo, Faculdade de Medicina da Universidade de São Paulo, São Paulo, SP, Brazil

Dear Editor,

Cancer of unknown primary origin (CUP) is a condition where metastatic cancer is diagnosed, but it is not possible to identify the primary tumor.^1^ Angiosarcoma is a group of vascular malignancies of endothelial cell origin.^2^ We report the case of a patient who was misdiagnosed with CUP in the acetabulum but with skin lesions that led to the diagnosis of metastatic epithelioid angiosarcoma.

A 57-year-old female presented ulcers on the vulva for three months. She had a past medical history of CUP affecting the acetabulum diagnosed two years before, treated with radiotherapy, with resolution of the lesion. She also had an asymptomatic complete aortic occlusion with collateral vascularization that remained stable for two years. The etiology for this occlusion was not found. She presented deep and painful ulcers with necrotic crusts affecting the vulvar and perianal areas (Fig. 1A). Skin biopsy showed neutrophilic lobular panniculitis, stains and cultures for bacteria, mycobacteria, and fungi resulted negative. Prednisone 1 mg/kg was started for a presumed diagnosis of pyoderma gangrenosum. After one month, no improvement was observed. Thalidomide 200 mg/day was introduced. After three weeks, livedoid areas on the tights and gluteal region were observed (Fig. 1B), and a biopsy showed an embolus of atypical epithelioid cells (Fig. 2A‒B). The emboli area was small, and immunohistochemistry was not able to evidence the phenotype of the neoplastic cells. Acetabulum biopsy was reviewed, the immunohistochemistry panel was expanded, and it showed positivity for CD31, FLI-1, ERG, INI-1, vimentin, AE1/AE3, and cytokeratin 7, and diagnosis of epithelioid angiosarcoma was made. Due to technical difficulties, the aorta lesion was not biopsied, but it was presumed to be the primary angiosarcoma site (Fig. 3A‒B). Treatment was started with paclitaxel. Livedo rapidly resolved, skin ulcers progressively healed (Fig. 4A‒B), and the aorta lesion presented a partial response. After one year of the end of treatment (seven cycles of paclitaxel), no evidence of disease was observed on the skin. However, after 14 months of the end of chemotherapy, progression of the disease was detected, with increased retroperitoneal lymph node. Chemotherapy with paclitaxel was restarted. After five cycles, partial response was achieved, but the renal function deteriorated progressively, and the patient died due to sepsis two years after diagnosis of angiosarcoma.

**Fig. 1:**
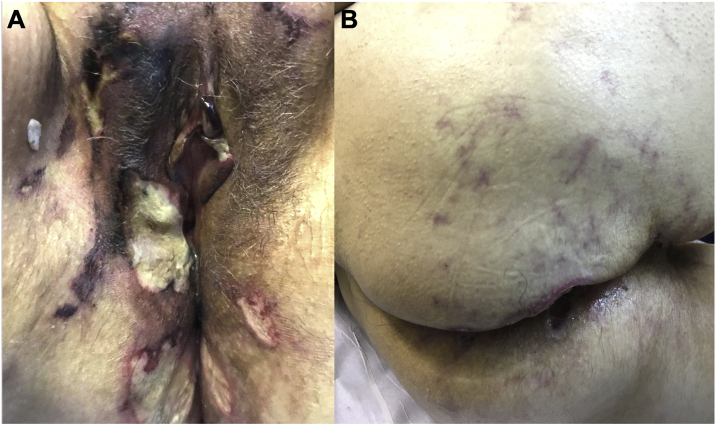
(A) Multiple deep ulcers with necrotic crusts and fibrin on vulvar area. (B) Livedoid lesions on the gluteal region.

**Fig. 2:**
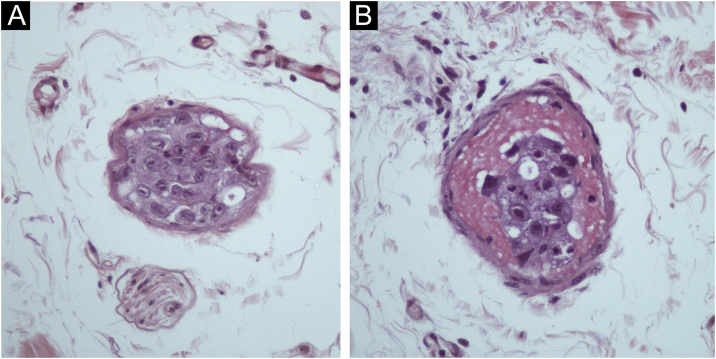
Emboli of atypical epithelioid cells on the skin (A and B, Hematoxylin-Eosin, 400×).

**Fig. 3:**
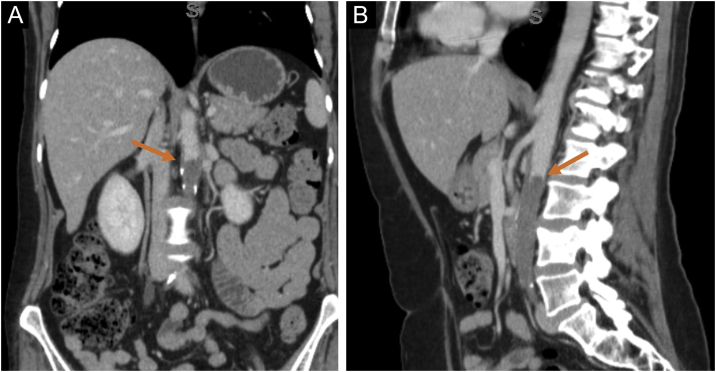
Computed tomography showing complete occlusion of the descending abdominal aorta (arrows). Coronal (A) and sagittal (B) scans.

**Fig. 4:**
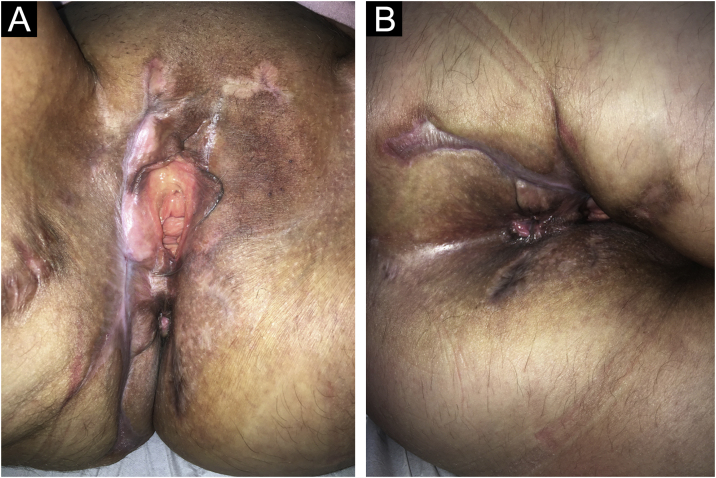
Complete healing of skin ulcers and livedoid lesions on the vulvar (A) and perianal (B) areas.

Skin is affected by metastasis of internal malignancies in 0.6%–10.4% of cancer patients. The most common primary sites of origin are breast (32.7%), lung (13.2%), oral cavity (6.2%), pharynx (6.2%), larynx (6.2%), colon and rectum (4.2%).^3^, ^4^, ^5^ Skin lesions are usually present as firm nodules affecting the trunk, head and neck. Involvement of genital and perineal areas is extremely rare.^3^, ^4^, ^5^

Angiosarcomas are rare and aggressive malignant vascular neoplasms of endothelial cell origin. Primary angiosarcoma is characterized by infiltrated angiomatous plaques or tumors in the cephalic region of elderly patients. Secondary angiosarcoma includes lymphedema-associated and radiotherapy-induced angiosarcomas.^6^ Epithelioid angiosarcomas are mostly observed in deep soft tissues.^6^, ^7^ Histopathology shows interanastomosing vessels lined by epithelioid endothelial cells and sheets of atypical cells. Immunohistochemistry shows positivity for vascular markers (CD31, CD34, ERG), cytokeratins, and CD30.^7^

Angiosarcomas originating from the heart and great vessels correspond to 3% of angiosarcomas.^8^ Metastases of aortic angiosarcomas are reported in 70% of the cases, and they are found in the bones, lungs, liver, skin, brain, bowels, and kidneys. Distal emboli may cause ischemia, leading to cyanosis, livedo, and necrosis.^9^, ^10^

In the present case, the patient was misdiagnosed with CUP affecting the acetabulum. The unusual dermatologic manifestation led to suspicion of infectious and inflammatory diseases, and the diagnosis of ulcers secondary to malignant emboli was confirmed after the development of livedoid areas. The investigation confirmed the diagnosis of angiosarcoma, and a more efficient tumor subtype-directed therapy was prescribed. This case highlights the importance of an extensive and persistent investigation of CUP cases to find the primary site, and the role of Dermatology was essential to conclude the correct diagnosis and prescribe the most appropriate treatment.

## Financial support

None declared.

## Authors’ contributions

Denis Miyashiro: The study concept and design; data collection, or analysis and interpretation of data; writing of the manuscript or critical review of important intellectual content; effective participation in the research guidance; intellectual participation in the propaedeutic and/or therapeutic conduct of the studied cases; critical review of the literature; final approval of the final version of the manuscript.

Regina Barros Domingues: Data collection, or analysis and interpretation of data; writing of the manuscript or critical review of important intellectual content; effective participation in the research guidance; intellectual participation in the propaedeutic and/or therapeutic conduct of the studied cases; critical review of the literature; final approval of the final version of the manuscript.

Malu Viter da Rosa Barbosa: Data collection, or analysis and interpretation of data; writing of the manuscript or critical review of important intellectual content; intellectual participation in the propaedeutic and/or therapeutic conduct of the studied cases; critical review of the literature; final approval of the final version of the manuscript.

Claudia Giuli Santi: Data collection, analysis and interpretation of data; writing of the manuscript or critical review of important intellectual content; intellectual participation in the propaedeutic and/or therapeutic conduct of the studied cases; final approval of the final version of the manuscript.

Marcello Menta Simonsen Nico: Data collection, or analysis and interpretation of data; writing of the manuscript or critical review of important intellectual content; intellectual participation in the propaedeutic and/or therapeutic conduct of the studied cases; final approval of the final version of the manuscript.

José Antonio Sanches: The study concept and design; effective participation in the research guidance; final approval of the final version of the manuscript.

## Conflicts of interest

None declared.
